# Impact of Integrated Care on the Rate of Hospitalization for Ambulatory Care Sensitive Conditions among Older Adults in Stockholm County: An Interrupted Time Series Analysis

**DOI:** 10.5334/ijic.5505

**Published:** 2021-06-09

**Authors:** Janne Agerholm, Antonio Ponce de Leon, Pär Schön, Bo Burström

**Affiliations:** 1 Aging Research Center, Karolinska Institutet, Stockholm, Sweden; 2 Center for epidemiology and community medicine, Stockholm, County Council, Sweden; 3 Department of Public Health Sciences, Karolinska Institutet, Stockholm, Sweden

**Keywords:** integrated care, Sweden, ambulatory care sensitive conditions, time series, aging, health care

## Abstract

**Introduction:**

Reducing avoidable hospital admissions is often viewed as a possible positive consequence of introducing integrated care (IC). The aim of this study was to investigate the impact of implementing IC in Norrtälje on the rate of admissions for ambulatory care sensitive conditions (ACSC).

**Method:**

Using interrupted time series analyses we investigated the effect of implementing IC in Norrtälje municipality in the northern part of Stockholm county, Sweden. The time period included 48 time points, from year 2000 to year 2011 with measurements before and after introducing IC in Norrtälje in 2006. In order to control for other extraneous events that could affect the outcome measure, but not related to the introduction of IC, we included a control population from Stockholm municipality.

**Results:**

After introducing IC in Norrtälje the rate of admissions for ACSC decreased. This decrease was greater in Norrtälje than in the matched control population, however the difference between the two areas was not statistically significant (p = 0.08).

**Conclusion:**

Introducing IC in Norrtälje may have had positive impact on admissions for ACSC for older people living in Norrtälje; however, the interpretation of the impact of IC on admissions for ACSC is complicated by intervening policy changes in health and social care during the study period.

## Introduction

The health problems of older people are often characterized by multi-morbidity and frailty in combination with multiple functional (physical and cognitive) limitations [1,2]. This results in increased needs of services from multiple providers, both in health and social care and often close family. Continuity, integration, and collaboration between health and social care providers are therefore essential if we are to meet the needs of an ageing population [3]. However, the current health and social care systems, both in Sweden and elsewhere, are fragmented with a focus on highly specialised services and therefore poorly designed to provide health and social care for patients with multiple health problems and social care needs [4]. Moreover, some groups of patients might have greater difficulties navigating in a fragmented and divided system, which introduces a risk of increasing inequalities in access and use of health and social care services.

Integrated care has become an international buzzword in the field of health policy and health management research as a way to tackle both financial and quality issues in a world with increasing costs and high demands on health and social care [5]. Integrated care systems have been advocated as a possible arena to reduce polypharmacy [6], to improve health-related quality of life, better functioning among multi-morbid and frail patients [7] and there are indications that rates of hospitalisation and readmission might improve [8]. However, robust evaluations of integrated care systems or models are rare [5,7,9].

In Sweden health care is decentralised and the responsibility of the 21 different regions. Social care on the other hand is the responsibility of municipalities. Both health- and social care have a public and private providers, however, both sectors are publicly funded and regulated. As every region is responsible for managing and prioritising resources for health care and every municipality for social care, care may vary between regions and municipalities (a further description if the Swedish health care system can be found in Anell et al. 2012 [10]).

This division of responsibilities between the regions and municipalities are not conducive to good coordination between health and social care. Therefore, policies to improve care coordination, especially for older people with complex health problems and severe needs, have been high on the social policy agenda in recent years. Integrated care projects of different scales have been one attempt to manage these problems in several regions of Sweden [11]. Nevertheless, the prime example of an integrated care model, is the TioHundra organisation in Norrtälje [12].

The company TioHundra is owned by the “Municipal association of health and social care in Norrtälje”, which is a cooperation between Region Stockholm (responsible for health care) and the municipality of Norrtälje (responsible for social care). This organisation was founded in 2006 after some years of political debate about whether the local hospital, owned and financed by the Region Stockholm, should be shut down due to financial difficulties. Huge protests from the citizens and the local politicians in Norrtälje led to a new initiative to improve health care and social care through extended cooperation between providers and a shared responsibility for both health and social care in one organisation.

TioHundra operates as one comprehensive health and social care organisation, owned, financed and managed jointly by the Norrtälje municipality and Region Stockholm, and was established with the objective to improve efficiency, quality and coordination in care provision while still controlling the costs. The process started in 2006 with the implementation of the macro structure and pooling of resources, but the implementation of full integration has been continuously improved along the way and is still ongoing (see ***Table 1***). The goal of the Norrtälje model is to achieve vertical and horizontal cooperation in order to better respond to the health care needs of the population. Besides shared financial responsibility between the municipality and the region for both health care and social care, the model is characterised by having a focus on health promotion for the population, as well as integrating the health and social care organisation on the administrative level. From the start TioHundra have particularly focused on interventions for older adults with complex health and social care needs [13]. A thorough description of the implementation of the integrated care model in Norrtälje can be found in Bäck & Calltorp from 2015 [13].

**Table 1:**
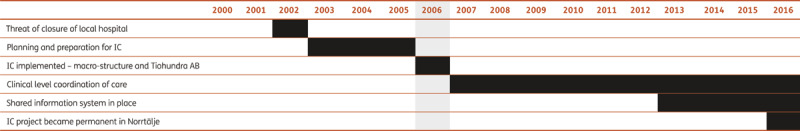
Timeline of the implementation of integrated care (IC) in Norrtälje.

The Norrtälje integrated care model has previously been evaluated regarding social care and how the staff experience the integration and coordination [14], but not substantively regarding health care utilisation. In theory, the integration of inpatient and outpatient hospital care, primary care and social care, as implemented in Norrtälje, might reduce the need for unplanned hospitalisation. Reducing avoidable hospital admissions is sometimes viewed as a possible positive consequence of introducing integrated care [5]. Ambulatory Care Sensitive Conditions (ACSCs) are conditions that with effective management and treatment should not lead to hospital admissions [15] and could be considered avoidable. High levels of hospital admissions for ACSCs are often used as an indicator of poor co-ordination within the health care system; especially between primary and secondary care; however, there are only a very few studies investigating how integrated care could influence hospitalisation for ACSC.

A study from Geneva, Switzerland, found that hospitalisations among frail older adults could be significantly reduced when recommendations for care were coordinated by a community geriatric unit in collaboration with the patient’s primary care physician and visiting nursing service. The control group were patients where care wewas left to the primary care physician and visiting nursing service, with no formal case management [16].

Another study from the UK, evaluating effectiveness of a multidisciplinary team case management model of high-risk patients, found no clinically significant changes for patients receiving the intervention [17].

Although very few studies have investigated the effect of integrated care on hospitalisation for ACSC, there are several studies on the effect of integrated care on hospital admissions in general. Two umbrella reviews focusing on patients with chronic conditions, including 50 [18] and 13 [19] systematic reviews and meta-analyses respectively, both found that integrated care reduces hospital admission for patients with chronic conditions, although the magnitude of the reduction could be questioned [18]. Another systematic review, focusing on older people in general, and not specific patient groups, also found a possible positive impact of integrated care on hospitalisation rates [9].

Lower socioeconomic groups have higher rates of ACSC [20]. This is primarily due to their higher disease burden, but a part of the differences could potentially be due to delay in seeking treatment, getting diagnosed or getting the right treatment [21]. When need is taken into account, several studies have shown that lower socioeconomic group do not utilise health care to the same degree as higher socioeconomic groups [22,23], especially among the older population [23]. In an integrated care setting with higher level of coordination between care providers it might be easier to meet the needs of more vulnerable groups and follow up on patients with more complex health care needs. Therefore, integrated care could be expected to have a differentially greater impact on rates of ACSC in lower socioeconomic groups and help increase equality in health care.

## Aim and research questions

The aim of this study was to investigate the impact of implementing integrated care in Norrtälje on the rate of hospitalization for ambulatory care sensitive conditions. More specifically we want to investigate:

- How does integrated care impact on rates of hospitalisation for ACSC among older people as compared with “standard care”?
- How does integrated care affect equity in rates of hospitalization for ambulatory care sensitive conditions among older people?

## Research Methods

The organizational changes in Norrtälje can be regarded as a natural experiment and will be evaluated using interrupted time series (ITS) analysis (14, 15). This is a quasi-experimental design with a series of periodic measurements before and after introducing IC in Norrtälje in 2006. The time period includes 48 time points, from year 2000 to year 2011.

In order to control for other extraneous events that could affect the outcome measure, but not related to the introduction of IC in Norrtälje, we included a control population from Stockholm municipality, individually matched by year, age, sex and level of income. Introducing control groups in interrupted time series analyses has been shown to increase validity of ITSs to a level comparable with randomized control trials [24,25].

### Data material

Data on hospitalisation for ACSC was obtained from the Region Stockholm administrative database for analysis and follow-up of healthcare utilisation (VAL). This database contains information on all registered outpatient and inpatient care financed by Region Stockholm.

Sociodemographic background characteristics were obtained from the ‘Longitudinal integration database for health insurance and labour market studies’ (LISA by Swedish acronym). The LISA database is a collection of individual level variables from different population registers linked individually through encrypted personal identity numbers. These data were individually linked to the VAL database by Statistics Sweden and used for matching purposes.

### Variables

The primary outcome variable is the quarterly rate of hospitalisation for ACSC. ACSC refers to hospitalizations for conditions that would have been possible to avoid by timely and effective use of primary care and includes both chronic diseases and acute diseases. The definition of ACSC may vary between different settings as well as the included conditions. In this study ACSC conditions were derived from the VAL register and coded using a categorisation adapted from that used by the British National Health Services [26]. The adapted categorisation of ACSC in this study have also been used in Finland [27,28]. ACSC in this study includes the following conditions: Acute Bronchitis, Angina, Asthma, Bacterial Pneumonia & Influenza, Chronic Obstructive Pulmonary Disease, Cellulitis, Congestive Heart Failure, Convulsions, Dehydration, Dental Conditions, Diabetes Complications, Epilepsy, Gangrene, Gastroenteritis, Hypertension, Immunization-Related and Preventable Conditions, Iron Deficiency Anaemia, Kidney and Urinary Tract Infections, Nutritional Deficiencies, Pelvic Inflammatory Disease, Perforated or Bleeding Ulcer, Severe ENT infection. The outcome was summed on area level and used in the interrupted time series as quarterly rates of ACSC per 10.000 inhabitants.

### Matching variables

The population in Norrtälje was slightly older, had a smaller proportion of men and on average a lower income than the control group. These factors have all been shown to be correlated with hospitalization for ambulatory care sensitive conditions. [20,29]. Therefore, age, sex and level of income was used to individually match the population in Norrtälje 1:1 with the control group.

When more than one individual in the comparison group matched the Norrtälje profile, then a random procedure was used to select one control individual. Age was used as a continuous variable. The income variable used was individualised disposable income (after taxes and transfers). Income was then categorised into income quintiles.

In the subgroup analyses on low income, only the lowest income group was used.

### Statistical analyses

We used the generalised additive model with the thin plate spline as a fitting method in the interrupted time series analysis to find the function best fitted for the data. Where the non-linear changes over time could be approximated by straight lines, we performed interrupted time series analyses using ordinary least squares to estimate the trends before 2006 as well as the trend change after. The Durbin-Watson test and the partial autocorrelation function were used to assess autocorrelation.

In order to assess whether changes in Norrtälje could be attributed to the introduction of IC we calculated quarterly rates of hospitalisation for ACSC in 2000–2011 for a matched population from Stockholm municipality. The relative difference between the two areas was then used to assess whether changes appeared over time between the two areas.

To investigate whether integrated care would affect equity in health care utilisation among the older population we calculated the relative index of inequality (RII) for hospitalisation for ACSC. RII was calculated based on the average cumulative proportion in each income quintile.

Data management was handled in SAS 9.4 and the interrupted time series analysis was performed in the statistical software R [30] using the package mgcv [31].

## Results

Norrtälje had 10,249 inhabitants in 2000 aged 65 years or above. In 2011 the number had increased to 13,893. In this period the mean age decreased by about 1 year from 75.7 to 74.7. The proportion of men increased from 45.3% to 47.5% and the proportion living alone decreased from 44.3% to 43.5% (see ***Table 2***).

**Table 2:** Demographic changes in Norrtälje from 2000 to 2011.


	2000	2001	2002	2003	2004	2005	2006	2007	2008	2009	2010	2011

**N**	**10249**	**10460**	**10660**	**10801**	**10921**	**11114**	**11330**	**11704**	**12242**	**12832**	**13103**	**13893**

**Age (mean)**	75.69	75.7	75.71	75.71	75.69	75.66	75.56	75.43	75.19	74.98	74.75	74.71

**Sex (%)**												

Men	45.29	45.38	45.54	45.72	45.83	46.07	46.44	46.69	46.8	47.11	47.15	47.47

**Living situation (%)**												

Cohabitating	55.75	51.63	52.14	51.28	54.69	54.67	54.43	54.94	55.61	56.11	56.45	56.55

Alone	44.25	43.3	43.02	43.74	45.31	45.33	45.57	45.06	44.39	43.89	43.55	43.45

Missing		5.08	4.84	4.98								


**Figure 1:**
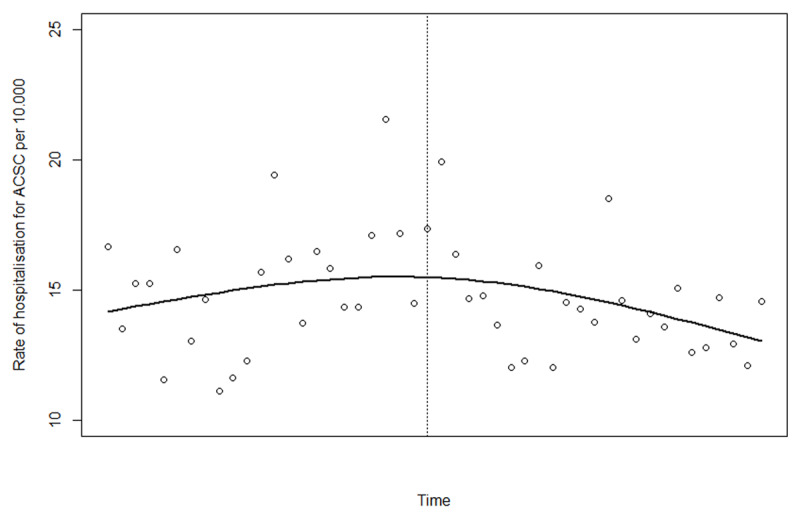
Results from the generalised additive model of the change in rate of hospitalisation for ACSC (The dots represent the actual observations).

**Figure 2:**
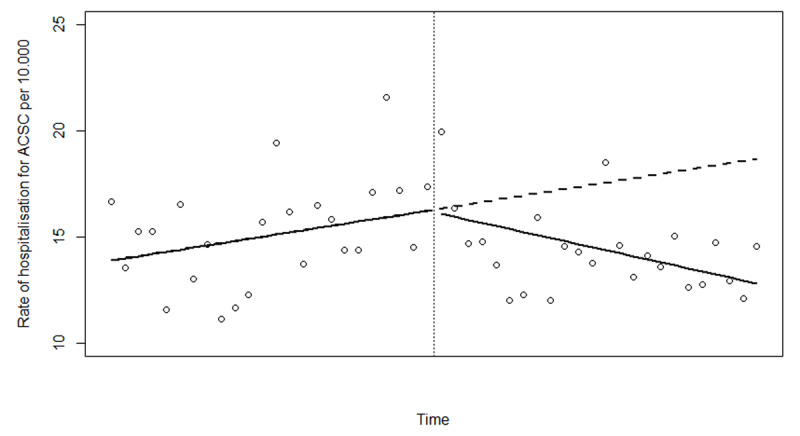
Linear trends in hospitalisation rates for ACSC before and after the introduction of IC in Norrtälje (The dots represent the actual observations).

**Figure 3:**
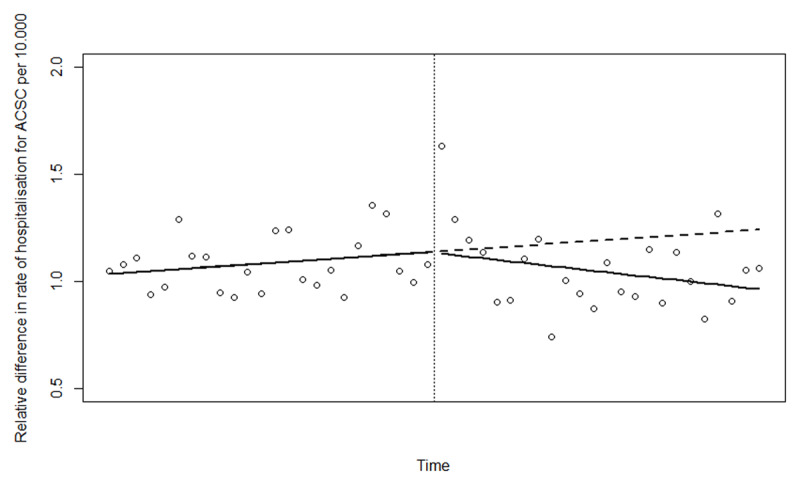
Trends in the relative difference in hospitalisation rates between Norrtälje and Stockholm before and after the introduction of IC (The dots represent the actual observations).

Based on Akaike information criterion (AIC) and evaluation of the results from the partial autocorrelation function (PACF) we found that a generalised additive model with a thin plate spline(?) and 3 degrees of freedom was the best fitted model for describing the non-linear change in rate of hospitalisation for ACSC (see ***Figure 1***, where the dots represent the actual observations and the line the estimates based on the fitted model). The effective degrees of freedom for the smoothing term was 1.8 and the model showed a nonlinear change that could be approximated by 2 straight lines; one before and one after the intervention. We therefore fitted a linear model using ordinary least squares giving the possibility to calculate linear trends before the introduction of IC as well as trend change after. The linear model showed that the rate of hospitalisation for ambulatory care sensitive conditions did decrease significantly after introducing IC in Norrtälje (see ***Figure 2***). When comparing to the changes in a control population this decrease was larger in Norrtälje, however not statistically significantly different from that in the control population (p = 0.08; see ***Figure 3*** and ***Table 2***).

For the age-group 80+ neither the pre-intervention trend nor the post-intervention trend was significant. The same was the case for the relative difference between Norrtälje and the control group. We did not find any significant changes in the relative index of inequality in Norrtälje in this time period (see ***Table 3***).

**Table 3:** Results from the linear regression models.


	TREND BEFORE IC		TREND CHANGE AFTER IC	
	
	VALUE	p-VALUE	VALUE	p-VALUE

**Rate of hospitalisation for ACSC**				

Population 65+ in Norrtälje	0.1018	0.0481	–0.2441	0.008

Population 80+ in Norrtälje	–0.0966	0.3748	0.2510	0.189

Relative Index of Inequality for the population of Norrtälje	0.0046	0.6027	–0.0021	0.892

**Relative difference in rate of hospitalisation for ACSC in Norrtälje compared to the matched control group**				

Population 65+	0.004	0.2478	–0.0117	0.083

Population 80+	–0.007	0.105	0.0096	0.184


**Figure 4:**
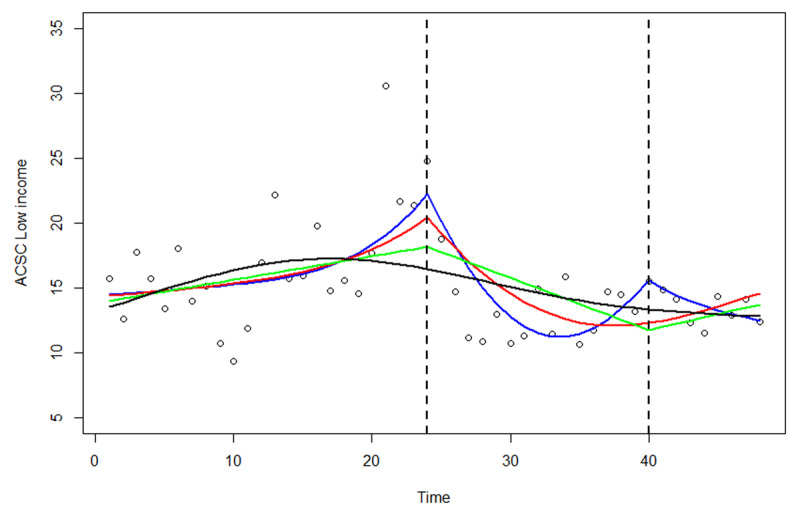
Results from the generalised additive model of the change in rate of hospitalisation for ACSC among the lowest income group (Black = Empty model; Red = adding a linear trend change after 2006; Blue = adding a second trend change after 2010; Green = Linear model with two trend changes using ordinary least squares; The dots represent the actual observations).

The change in rate of hospitalisation for ACSC among the lowest income group was best fitted with a generalised additive model (GAM) with a thin plate spline and 4 degrees of freedom and could not be approximated by two straight lines to calculate the trend and trend change after the introduction of IC. The predicted line based on the GAM without fixed effects is shown in ***Figure 4*** (black line). Adding a linear trend term from 2006 decreased the AIC and showed a significant decreasing post-intervention trend (red line in ***Figure 4***). The best fitted model was a GAM with two trend terms one post-intervention trend and one post-2010-trend (blue line). This model showed a steep decreasing post-intervention trend from 2006 and a decreasing, but much less steep decreasing trend from 2010 (both terms were significant; see ***Table 4***). The green line shows the fitted line from a linear model with two added linear trend changes in 2006 and 2010 (only 2006 was significant).

**Table 4:** Results from the generalised additive model and a linear model of the rate of hospitalisation for ACSC in Norrtälje among individuals with low income. The results correspond to the lines in ***Figure 4*** in the following way: GAM model 1 = black line; GAM model 2 = red line; GAM model 3 = blue line; Linear model = green line.


	GAM MODEL 1		GAM MODEL 2		GAM MODEL 3		LINEAR MODEL	
			
	VALUE	p-VALUE	VALUE	p-VALUE	VALUE	p-VALUE	VALUE	p-VALUE

Intercept	15.11	<0.001	27.60	<0.001	38.643	<0.001	13.82	<0.001

Time	n.a.		n.a.		n.a.		0.182	0.056

Trend 2006			–1.999	0.005	–3.527	<0.001	–0.583	0.005

Trend 2010					–1.983	0.016	0.645	0.155

AIC	268.27		260.48		254.96		267.31	


### Sensitivity analyses

Two major changes to health and social care policy were implemented in the post intervention period. In 2009 a choice reform in social care was implemented, introducing free choice of provider in social care in the entire Stockholm region, coupled with the right to free establishment for private providers of social care. This led to multiple providers establishing in Norrtälje alongside Tiohundra, challenging the coordination between health and social care. In 2010 a similar reform was introduced in primary care giving private providers of primary care the right to establish in Norrtälje, In the rest of Region Stockholm the reform in primary care was implemented already in 2008. However, in Norrtälje, Tiohundra was still, by far, the main provider of both health and social care. Due to these policy reforms, we made sensitivity analyses introducing the possibility of additional trend changes in 2009 and 2010 for Norrtälje and 2008 and 2009 for the Stockholm control group.

For most outcome measures, additional trends were not significant and did not add to the interpretation of the results. However, introducing an additional trend change in 2009 did make the relative changes between Norrtälje and Stockholm significant. To investigate this further, we therefore restricted the analyses to three years pre and three years post IC, in order to minimise the effect of additional policy changes.

For the rate of ACSC in Norrtälje the coefficients were in the same direction as the overall analysis. The pre-intervention trend was not as steep (0.0464 vs 0.1018), however the post-intervention trend change was somewhat larger (–0.3856 vs –0.2441). None of the estimates were statistically significant in the model based on data from 2003–2008.

When analysing the relative difference between Norrtälje and the matched control group, results were in the same direction. The pre-intervention trend in the matched control group was 0.0133 (p = 0.282) and the trend change after the intervention was –0.0415 with a p-value of 0.056. This indicates that in Norrtälje, the rate of hospitalisation for ACSC decreased more in comparison with the matched control group in the first years after IC was implemented. This corresponds to a 36% decrease in the relative difference in 2008 compared to the difference that would have been, had the pre-intervention trend continued.

## Discussion

In this study we have examined the impact on rates of ACSC following the organisational change in health and social care in Norrtälje that started 2006 with the foundation of the company TioHundra. The goal was to achieve vertical and horizontal cooperation in order to better respond to the health care needs of the population in Norrtälje by pooling resources from the municipality of Norrtälje and Region Stockholm and integrating health and social care into one organisation. The results showed that the rate of hospitalisation for ACSC did drop among the older population living in Norrtälje after introducing IC in Norrtälje. The decrease in the rate of hospitalisation for ACSC was greater in Norrtälje than in a matched control group, however not significantly different from the decrease in the matched control group (p = 0.083). Looking at only the period immediately after the introduction of IC the decrease was more pronounced (p = 0.056). Other policy changes that were introduced after the implementation of IC in Norrtälje may have interfered with the decreasing trend in Norrtälje. It might have become more difficult to maintain good co-ordination and integration between multiple providers in the years after introducing free establishment for private providers of health and social care in Norrtälje.

For the group aged 80+ years there was a tendency to an upward trend change, however neither the pre-intervention trend nor the post-intervention trend change was significant, indicating that the rate of hospitalisation for ACSC was stable in this age group. These results are surprising as TioHundra have had a specific focus on the frail and older population of Norrtälje and we would have expected this group to have benefitted more. At the same time studies and reports suggest that older people and people suffering from multimorbidity have benefitted less from the primary care reform and receive relatively less primary care compared to other patient groups [32,33], which could influence the results.

The trends in the group with low income could not be modelled in a linear model with a pre-intervention trend and a post-intervention trend change. Instead, we used the GAM and found that a model with two post-intervention trends, one from 2006 and one from 2010, best fitted the data. The results of this model suggested a decrease in rate of hospitalisation for ACSC in 2006 and a decrease, but less steep, in 2010.

### Strengths and limitations

Robust evaluations of comprehensive integrated care systems are extremely rare and in many cases programmes are evaluated using uncontrolled before and after comparisons [5].

One strength of the approach in this study is the long time-series we were able to create using the unique registers in Sweden and the possibility to monitor changes in outcomes several years before and after introducing the integrated care model. On the other hand, a weakness of this longitudinal design is that it introduces a risk of other events influencing the outcome, such as the policy reforms in health and social care. Including a control group from an area close to Norrtälje, bound by the same rules and regulations, limits these threats to validity, as general changes in e.g. rules and regulations will also be detected in the control group. This makes it more reliable that the detected differences could be attributed to the different level of integration in the organizations.

Nevertheless, some policy changes were not introduced at the same time in Norrtälje and the control area. The primary care reform, introducing a new reimbursement system, free choice of provider as well as free establishment for private providers were introduced in Region Stockholm, except Norrtälje, in 2008. In Norrtälje this reform was not introduced until 2010. It is unclear what impact the time difference in introducing the reform could have had on the results of this study. The reform increased the number of providers of primary care in many areas [34] as well as the number of visits to primary care [32]. The two years difference in introducing this reform may have affected the results in this study, as the potentially positive effects on hospitalisation for ACSC might have been introduced later in Norrtälje and not captured by the models in this study. The sensitivity analyses suggest that, when trying to exclude the effect of the reforms by restricting the number of years post the intervention, Norrtälje did differ from the control group, however, more studies are needed in order to fully understand the impact of the reforms.

In what we refer to as standard care, health care and social care are separated and financed by the regions and the municipality, respectively. There is no shared funding or responsibility between the two organisations and there are no financial incentives for e.g. social care to minimise hospital care or vice versa as these are two separate budgets and two different organisations responsible for each sector. Nevertheless, as many patients move between e.g., hospitals and social care, there are of course routines for how transitions between the two systems should work. Even in standard care there is a desire to make this transition as smooth and positive for the patient as possible and e.g., give the necessary information to the social care system, when discharging a patient from inpatient care. This could be a reason for the modest change we see in this study.

We have hypothesised that living in an integrated care system with better possibility for communication between health care providers, as well as social care providers, potentially could lead to quicker attention to older people’s health care needs and thereby avoiding hospitalisation for conditions that could have been attended in primary care. In this study we have looked at a long range of conditions; however, previous studies have suggested that it might only by some of the ACSC that are related to quality and continuity in primary care [35]. As Norrtälje is a relatively small municipality, breaking down the number into different conditions made the variation between years much larger and gave room for outliers and empty cells to have too large impact on the results.

The fact that the implementation of IC in Norrtälje started with a focus on the macro structure and pooling of resources, and that the process of getting to full integration has been a longer process, has made the evaluation difficult and might be one reason for the modest changes that we see in this study. Longer follow-up times introduce the risk of other factors influencing the outcome, as has been the case for this study. The assumption for the interrupted time series analysis is that only the intervention affects the post intervention trend change, and therefore it can be estimated with only one estimate. Nevertheless, if other factors are introduced after the introduction of the initial intervention, there needs to be more interruptions in the model to allow for these changes as well. That might be the reason for the fact that we find a steeper decrease in Norrtälje compared to the control group in the period immediately after introducing IC. Studying the effect close in time to the intervention, there is a higher chance of capturing the pure effect of the intervention.

IC is a broad concept and integration can be achieved in different ways and on different levels. To the best of our knowledge this is the first study to investigate the effect of organisational integration between health and social care on the rate of hospitalisations for ACSC. The lack of possibility to compare to previous studies makes it difficult to understand whether these results can be generalised for other IC organisations or whether they are specific to the way health and social care is integrated in Norrtälje. Further research is needed to confirm our results.

The one study we found with positive effects of IC on hospitalisations for ACSC focused on frail older people [16]. In this study, we have focused on the general older population, both individuals with high health care consumption and individuals with no or very little health care consumption. The effect of the intervention in Norrtälje might be more profound for patient groups with higher health care consumption. Further research is needed to understand how IC affects different patient groups regarding hospitalisation for ACSC.

## Conclusion

After introducing IC in Norrtälje the rate of hospitalisation for ACSC decreased among the older population living in Norrtälje. This decrease was greater than in a matched control population in an area with standard care, however the difference between the two areas was not statistically significant (p = 0.08). Analyses of changes in the relative index of inequality in hospitalisations for ACSC showed no significant changes over time, indicating that IC did not affect inequality in hospitalisation for ACSC. The interpretation of the impact of IC on hospitalisation for ACSC is complicated by intervening policy changes in health and social care during the study period.
